# Biocatalytic Production of 2-α-d-Glucosyl-glycerol for Functional Ingredient Use: Integrated Process Design and Techno-Economic Assessment

**DOI:** 10.1021/acssuschemeng.1c07210

**Published:** 2022-01-11

**Authors:** Andreas Kruschitz, Bernd Nidetzky

**Affiliations:** † Austrian Centre of Industrial Biotechnology (acib), Krenngasse 37, 8010 Graz, Austria; ‡ Institute of Biotechnology and Biochemical Engineering, Graz University of Technology, NAWI Graz, Petersgasse 12, 8010 Graz, Austria

**Keywords:** advanced biomanufacturing, integrated process design, techno-economic assessment, 2-α-d-glucosyl-glycerol, sucrose phosphorylase, waste prevention

## Abstract

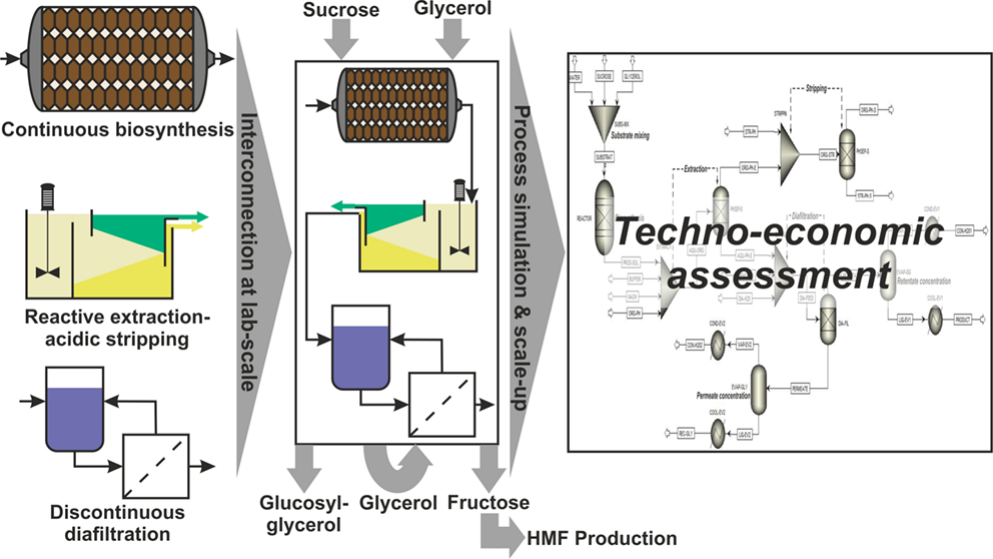

Advanced biomanufacturing builds on production processes that are both profitable and sustainable. Integrated design of process unit operations, geared to output efficiency and waste minimization and guided by a rigorous techno-economic assessment, is essential for development aligned to these central aims. Here, we demonstrate such a development for the biocatalytic production of the biological extremolyte 2-*O*-α-d-glucosyl-glycerol (2-GG) for functional ingredient application. The process was aligned in scale over all steps (∼180 g product; ∼2.5 L reaction mixture) and involved continuous enzymatic synthesis from sucrose and glycerol interlinked with reactive extraction and nanofiltration for product isolation (purity of ∼80 wt %) and side stream recovery. Glycerol used in ∼6-fold excess over sucrose was recycled, and hydrothermal conversion into 5-(hydroxymethyl)furfural was evaluated for the fructose by-product released from sucrose. Based on a process mass intensity (total mass input/mass product) of 146, ∼80% of the total mass input was utilized and an *E*-factor (mass waste/mass product) of 28 was obtained. EcoScale analysis revealed a penalty point score of 44, suggesting an acceptable process from a sustainability point of view. Process simulation for an annual production of 10 tons 2-GG was used for the techno-economic assessment with discounted cash flow analysis. The calculated operating costs involved 35 and 47% contributions from materials and labor, respectively. About 91% of the material costs were due to chemicals for the reactive extraction-acidic stripping step, emphasizing the importance of material reuse at this step. Glycerol recycling involved a trade-off between waste reduction and energy use for the removal of water. Collectively, the study identifies options and boundaries of a profitable 2-GG process. The minimum selling price for 2-GG was calculated as ∼240 € kg^–1^ or smaller. The framework of the methodology presented can be generally important in applied bio-catalysis: it facilitates closing of the gap between process design and implementation for accelerated development.

## Abstract 2

An integrated process for the production of 2-*O*-α-d-glucosyl-glycerol was demonstrated, and its sustainable performance was revealed by a detailed techno-economic assessment.

## Introduction

Integration is a leading principle of process development in advanced biomanufacturing. It implies production processes that integrate the individual up- and downstream processing steps into a holistic process entity.^1,2^ Integration involves coordination and interconnection of process steps not only to enable efficient production of the desired compound but also to recover side streams for further utilization.^3^ While individual process steps are often analyzed in minute detail, less attention is paid to the crucial point of integration. Since individual process steps can have profound influence on each other, their assembly into a complete process requires careful assessment in an interactive process network type of approach.^4^ Rigorous categorization of process options for targeted development builds on the data from techno-economical evaluation in two phases.^5^ First, technical performance is examined for the process design(s) considered over all steps carried out at the coordinated scale. Second, process modeling is done for production at the scale envisioned for manufacturing.^6^ This provides the relevant mass and energy balances and enables a rough sizing of the required equipment. Capital and operation costs can thus be estimated to evaluate process feasibility and profitability.^7^ Analysis in the way described gives a sound basis for process decision making to guide the process development toward implementation. Process steps representing bottlenecks are identified, and their critical interactions in the overall process network are revealed.^7,8^ In the field of applied bio-catalysis, the importance of such an integrated development is widely recognized, yet the literature demonstrating it in detail is scarce. Process modeling with accompanied techno-economic analysis is often limited by experimental data from a dedicated performance evaluation of the entire process.

**Scheme 1:**
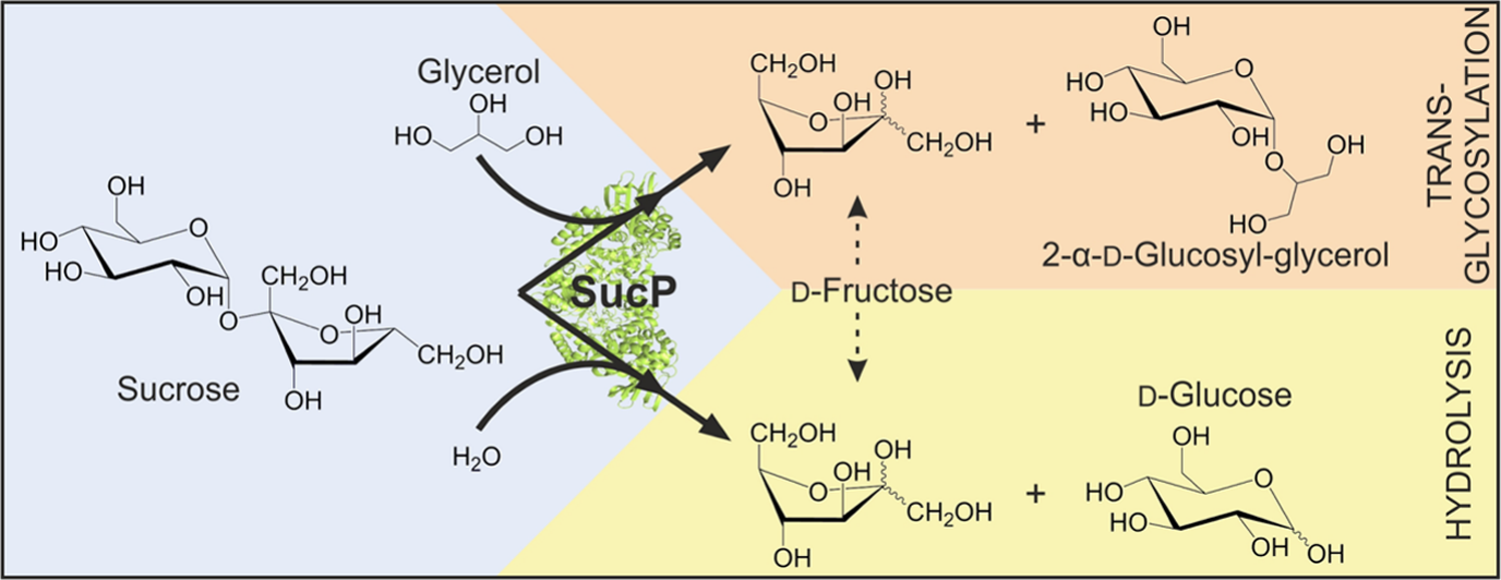
Reaction of SucP to Synthesize 2-α-d-Glucosyl-glycerol from Sucrose and Glycerol Hydrolysis is a minor side reaction when glycerol is used in excess. The figure was adapted from Kruschitz et al. 2021.^16^

Here, we show integrated process development according to the approach outlined above for the biocatalytic production of 2-α-d-glucosyl-glycerol (2-GG).^9−12^ 2-GG is a natural extremolyte found in plants and microorganisms. It is commercially produced for active ingredient use in cosmetics. Another promising use of 2-GG is that of a prebiotic in food and feed products. 2-GG is synthesized industrially from sucrose and glycerol by sucrose phosphorylase (SucP)-catalyzed transglycosylation, as shown in Scheme 1.^13^ The used enzymes are stereospecific and show high regioselectivity on glycerol (>80% 2-GG).^14^ The glycerol substrate is applied in excess (up to six-fold) for two reasons. First, sucrose conversion is driven to completion. The sucrose-free reaction mixture is preferred for the recovery of the 2-GG product in application-grade purity, as discussed later. Second, glycerol competes with water for reaction with SucP. Therefore, sucrose hydrolysis is suppressed at high glycerol concentration (Scheme 1).^15^ The product solution thus mainly contains 2-GG, the by-product fructose and unconverted glycerol. Its composition defines the main tasks of an efficient downstream processing aimed at product isolation and waste reduction in suitable combination.

**Figure 1:**
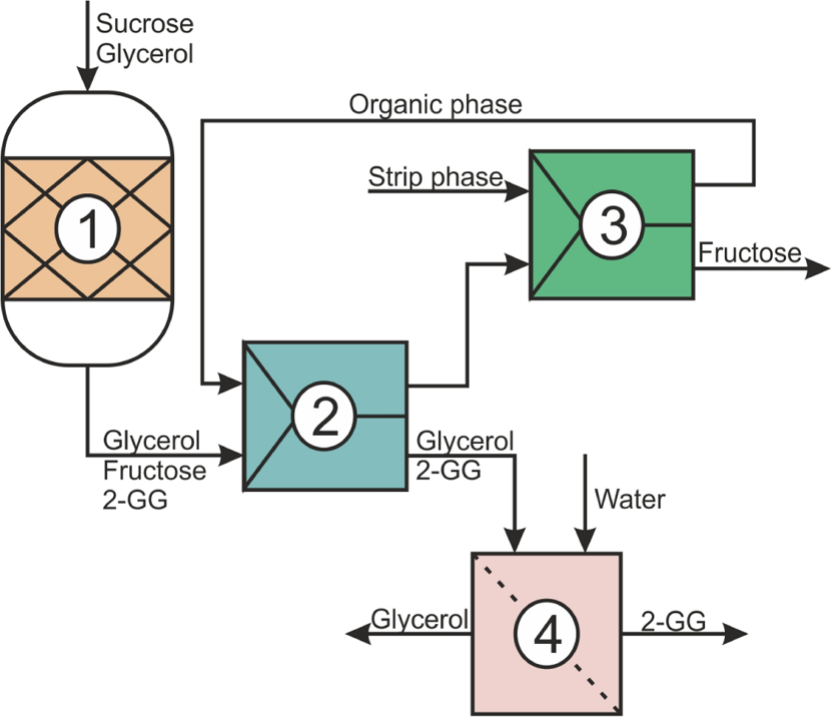
Lab-scale process for 2-GG production, including (1) continuous bioconversion, (2) reactive extraction, (3) acidic stripping, and (4) discontinuous diafiltration.

The upstream part of the considered 2-GG process (Figure 1) is performed continuously. A solid preparation of SucP is used as the catalyst. *Escherichia coli* cells that express the recombinant enzyme in high amounts are immobilized through entrapment in the polyacrylamide (PAM) matrix. Flow synthesis in a packed-bed reactor achieves high productivity while it still meets the high demand in sucrose conversion.^16^ Process technologies that accomplish individual tasks of the downstream processing have been proposed. Glycerol is removed by multistep discontinuous diafiltration.^17^ Fructose is separated through a three-step reactive extraction-acidic stripping process.^18^ From these earlier studies, a promising structure of the integrated 2-GG process is shown in Figure 1. However, the different process steps have not been shown at a coordinated scale and interlinked into a complete production process. Moreover, the idea of using the process side streams exists only as concepts. Recycling of residual glycerol for 2-GG synthesis has not been evaluated, and the usage of the recovered fructose in further upgrading processes remains to be demonstrated. Process modeling and techno-economic analysis cannot be performed without experimental validation of the proposed production. The current study was performed to assess the technical feasibility, and the economic profitability in principle, of the integrated 2-GG process. Detailed techno-economic analysis characterizes the process in terms of the cost structure as well as the cost contribution of the individual unit operations. This identifies critical process boundaries and shows options for further optimization. For the fructose recovered from the process, conversion into the base chemical 5-(hydroxymethyl)furfural (HMF) was evaluated. HMF is an important platform chemical, and fructose is a well-suited substrate for its synthesis.^19−21^ The route to HMF contributed to complete raw material utilization and process waste reduction. It explores an important non-food use for the fructose released during 2-GG production. Additionally, green process metrics, such as environmental factor (*E*-factor), process mass intensity (PMI), and EcoScale, were calculated to assess the environmental impact of the 2-GG process. The *E*-factor and the PMI are defined as the ratios of mass waste/mass product and total mass input/mass product, respectively.^3,22^ The EcoScale was first introduced in 2006 by Van Aken et al.^23^ This analysis method compares the considered process with an ecologically ideal process. All three metrics are simple to calculate, well accessible, and comprehensible. Therefore, they are well-suited to analyze the eco-credentials of a process at an early development stage. Overall, this study of 2-GG production can be generally important in applied biocatalysis. A framework of the methodology is presented that facilitates a closing of the gap between design and implementation of the economically viable and sustainable bioprocess. This is critical to accelerate the development and strengthens of the competitiveness of biocatalysis in the industrial production of chemicals.

## Experimental Section

### Chemicals

Acrylamide and 3-(dimethylamino)propionitrile were obtained from Sigma-Aldrich (St. Louis, Missouri, USA). *N*,*N*′-Methylenebis(acrylamide) was obtained from Carl Roth (Karlsruhe, Germany). Potassium persulfate was obtained from Merck (Darmstadt, Germany). Naphthalene-2-boronic acid was obtained from Matrix Scientific (Columbia, South Carolina, US), and Aliquat 336 was obtained from Thermo Fisher Scientific (Kandel, Germany). Glycoin natural (52.8 w% 2-GG) was obtained from bitop AG (Dortmund, Germany). Other chemicals were obtained from Carl Roth, Merck, or Honeywell (Charlotte, North Carolina, USA).

### Preparation of the Immobilized Catalyst

*E. coli* BL21 (DE3)-agp (plasmid pQE30) was employed to produce BaSucP_P134Q (N-terminal His-tag), a SucP variant from *Bifidobacterium adolescentis*.^14^ Bioreactor cultivation was done at 37 °C in a Biostat CT (5 L) system (B. Braun Biotech International, Germany) equipped with a Biostat C controller (pH 7.0; air flow rate 7.5 L min^–1^; ∼40% air saturation). The protocol was adapted from the literature.^24^ Expression was induced with 0.25 mM IPTG, which was added at OD 2.0–3.0, followed by overnight incubation at 25 °C. Ampicillin (115 mg mL^–1^; 5 mL) was added before inoculation and induction. Cells were grown to ∼30 g wet mass L^–1^ and harvested with a HiCen SR ultracentrifuge (Herolab, Wiesloch, Germany; 4 °C, 5000 rpm, 20 min). The cell pellet was frozen at −21 °C and thawed to permeabilize the cells. The thawed cells were encapsulated in PAM using a reported procedure.^16,25^ Briefly, cells were thoroughly resuspended in 100 mM HEPES buffer (pH 7.0) at a cell loading of 0.5 g wet cells mL^–1^. Acrylamide was dissolved in the cell suspension at 0.1875 g mL^–1^, and *N*,*N*′-methylenebis(acrylamide) (10 mg mL^–1^) and 5 v% 3-(dimethylamino)propionitrile solution (0.125 mL mL^–1^) were admixed. For polymerization, 2.5 wt % potassium persulfate (0.125 mL mL^–1^) was used for 30 min on ice. The resulting rigid PAM immobilizate (PAM-I) was shredded with a scalpel and a hand blender for some seconds. The PAM-I particles were sieved through 0.25 and 2.00 mm sieves and were subsequently thoroughly washed with water. The specific activity of PAM-I particles in continuous operation (bed volume ∼40 mL) was estimated to be around 3–6 μmol 2-GG min^–1^ g^–1^.

### Lab-Scale Process for 2-GG Production

#### Synthesis

This was carried out continuously in a packed-bed reactor scaled up to ∼400 mL working volume. A XK50/30 column (ID 50 mm, GE Healthcare, Chicago, Illinois, USA) was filled with PAM-I particles in the size range of 0.25–2.00 mm. The bed height was ∼20.3 cm. The column was connected to a circulating water bath for temperature control at 40 °C.^16^ Feed was from an Azura P4.1S pump (Knauer, Berlin, Germany). The packed bed was washed with water and substrate solution (0.35 M sucrose, 1.9 M glycerol) at 1 mL min^–1^. Synthesis was done with a substrate delivered at 3 mL min^–1^ (space velocity of 0.45 h^–1^) for ∼16–17 h.

#### Extraction and Stripping

The previously reported three-step extraction, based on a mixer-settler setup, was applied.^18^ The whole process was split into six batches. For each batch, 0.42 L of the reaction mixture was diluted with 0.73 L 0.3 M Na_2_CO_3_/NaHCO_3_ buffer (pH ∼ 10.6) and mixed with 1.15 L organic phase, made of octanol/heptane (4/1, v/v) containing ∼17 g L^–1^ naphthalene-2-boronic acid and ∼75 g L^–1^ Aliquat 336. The organic phase was stripped with 0.22 L nitric acid (∼8.6 wt %), recycled, and mixed again with the already extracted aqueous phase. The whole procedure was carried out two more times. In-between the extraction steps, the pH of the aqueous phase was adjusted to ∼10.5 with 10 M NaOH. Mixing was performed with a RZR 2020 overhead stirrer (Heidolph, Schwabach, Germany) for 5 min at ≥800 rpm, and the phases were separated in a 2 L separation funnel after 5–10 min.

#### Discontinuous Diafiltration

The aqueous phase leaving the extraction/stripping process was ultrafiltered through a 0.45 μm filter and its pH was adjusted to ∼8 with conc. HCl. This was only necessary to avoid damaging of the membrane. Multicycle discontinuous diafiltration of the aqueous phase was performed with the automated lab-scale filtration unit Memcell (Osmo Membrane Systems, Korntal-Münchingen, Germany). A type DL 1812 spiral wound membrane (0.32 m^2^) from SUEZ (Trevose, Pennsylvania, USA) with a molecular mass cut-off of 150–300 Da was applied, which was conditioned up to 25 bar prior to use. Of note, the Memcell and the membrane-module comprised a dead volume of ∼1 L, which is filled with water. This caused a dilution of the feed solution. The diafiltration was carried out at constant permeate flux (∼10 kg m^2^ h^–1^), 30 °C and a flow rate in the membrane module of ∼350 L h^–1^. In each diafiltration cycle, the feed was concentrated by a factor of ∼2.75. The resulting retentate was diluted with water to the original feed volume, circulated in the Memcell for ≥10 min without an applied pressure to properly mix the solution, and then the next diafiltration cycle was started. The discontinuous diafiltration was carried out until the glycerol content in the retentate was <1 g L^–1^.

### Glycerol Recycling

Permeate (200 mL) from the diafiltration was concentrated to ∼15 mL with a Heidolph Laborata 4000 rotary evaporator (Schwabach; Germany) equipped with a Vacuubrand PC 2001 vacuum pump (Wertheim, Germany) at 80 mbar and 60 °C. The concentrated permeate was mixed with fresh glycerol solution (160 g L^–1^) at a ratio of ∼2.25/1.00 (v/v), and then 0.3 M of sucrose was admixed. Sucrose conversion was carried out in 2 mL volume at 40 °C on a Thermomixer comfort (900 rpm; Eppendorf AG, Hamburg, Germany). PAM-I particles (50 mg) were added to the reaction solution. Samples were drawn at certain times and heat-treated at 99 °C for 5 min.

### Analytics

HPLC analysis of the lab-scale process samples was performed with a Shimadzu LC-20AD system (Kyoto, Japan) connected to an autosampler (SIL-20AC HT) and a RI-detector (RID-20A). A YMC-Pack Polyamine II/S-5 μm/12 nm column (250 × 4.6 mm) (YMC, Kyoto, Japan), a Bio-Rad Aminex HPX-87C column (300 × 7.8 mm), or a HPX-42C column (300 × 7.8 mm) (Bio Rad, Hercules, US), all equipped with a guard column (20 × 4.0 mm for YMC, 30 × 4.6 mm for Aminex), were used. The eluent was 75/25 acetonitrile/water (YMC) or water (Aminex). Operation conditions were 1 ml min^–1^, 25 °C, and 30 min for the YMC column; 0.5 ml min^–1^, 80 °C, and 25 min for the HPX-87C column; and 0.6 mL min^–1^, 85 °C, and 25 min for the HPX-42C column. The sample volume was 20 μL.

### Techno-Economic Assessment

#### Process Design and Simulation

The process shown in Figure 1 was modeled with Aspen Plus V11 (Aspen Technology, Bedford, Massachusetts, USA). Compounds were imported from databases or were user-defined according to the physicochemical properties estimated with NIST ThermoData Engine. Compounds (e.g., organoboronic acid, Aliquat 336) not described by either methods were implemented as the Aspen pseudo-component. NRTL was used as a property method. Note: the usage of the compounds not available in the Aspen Plus databases did not limit the simulations. Specifications of the individual process steps were extracted from the experimentally measured data and are summarized in Table S1. Therefore, the simulations shown are not only based on the equations of state as implemented in Aspen Plus.

#### Economic Assessment

The 2-GG process was considered to be integrated in an existing biotechnology company located in Middle Europe. Calculations are in EUR € (1.00 € ≙ 1.22 US$). Project costs were divided into total capital investment (TCI) and operating costs (OC). The TCI, the sum of fixed capital investment (FCI) and working capital (WC, 5% of FCI), was calculated as already presented in the literature.^26−30^ The FCI, including total direct (TDC) and indirect (TIC) costs and other capital costs (OCC), was estimated based on the total equipment costs (TEC). The TEC were determined with the Aspen Plus Economics package. Exceptions were the costs for ultra- and diafiltration units, which were estimated from manufacturer quotes. The TDC (installation, piping, instrumentation, insulation, electrics, yard improvement, and auxiliary facilities), the TIC (engineering and construction), and the OCC (contractor’s fee and contingency) were estimated to be 250, 125, and 60% of the TEC.

The OC comprised variable (VOC) and fixed (FOC) operating costs.^26,27^ The estimation of VOC, including costs for raw materials, utilities, and waste management, was based on the mass and energy balances of the Aspen Plus simulation. Waste for disposal was divided in four categories, namely, water, aqueous, hazardous, and solid waste.^30,31^ All prices used for the calculations of VOC were extracted from the literature or based on manufacturer quotes (Table S2). If variable prices were found, the (reasonable) mean was taken. FOC contained labor costs, labor burdens, maintenance, insurance, and overhead costs (accounting for marketing, managing, etc.). The labor costs were calculated from the annual salaries (taken from Austrian wage agreements for the chemical industry) of the employed staff (Table S3). The labor burden was assumed to be 80% of the annual salaries. Maintenance was 3% of TEC and TDC, insurance was 1% of FCI, and overhead costs were 5% of OC.^26,27^

#### Discounted Cash Flow Assessment

To assess the profitability of the process, the process costs (i.e., TCI and OC) and the process revenues were compared with each other in a discounted cash flow assessment (DCFA).^26^ It was assumed that OC and revenues increase by 2% each year. The equations and parameters used for the DCFA can be found in the Supporting Information (Table S4). The minimum selling price (MSP) was calculated for a net present value of zero to assess the profitability of the process.^26,27,29,30^ A sensitivity analysis was performed to examine if process and economic fluctuations affect the profitability of the process.^26,27,29^

## Results and Discussion

### Lab-Scale Process for 2-GG Production

Figure 1 shows the core structure of the production process. Although individual processing steps have been studied before,^16−18^ the integrated process is only shown from the current study. Additionally, the analysis of unit operations aligned in the scale involved a scale up by 10-fold or more compared to earlier studies. The mass balance data thus obtained are the basis for process simulation and techno-economic analysis. The developed 2-GG process had two main targets: (1) to produce a 2-GG solution with a glycerol and fructose content of <2 and <10 wt % based on 2-GG and (2) to recover glycerol and fructose for further utilization. Tasks of the individual processing steps, as mentioned later, are relative to these targets.

#### 2-GG Synthesis

The feed solution contained 118 g L^–1^ sucrose and 177 g L^–1^ glycerol. The continuous operation of the packed-bed reactor filled with the PAM-I particles was stable and consistent. No visible change of the packing (e.g., compression and channeling) was observed at the space velocity of 0.45 h^–1^. Within 14 h, 2.52 L of reaction solution was produced. The sucrose conversion was ∼97%. The concentrations of 2-GG, fructose, and glycerol were 73, 62, and 145 g L^–1^, respectively. This corresponds to a 2-GG molar selectivity based on fructose of 84% (see Scheme 1). Glucose was formed at ∼3 g L^–1^ and 1-GG at ∼10 g L^–1^, corresponding to a regioselectivity for the favored 2-GG of ∼88%.

#### Extraction and Stripping

The reaction solution was diluted (∼2.75-fold) with buffer (pH ∼ 10.6) to give the aqueous phase. It was fed in the three-step reactive extraction-acidic stripping cascade, which comprised alternating extraction and stripping steps, to separate the fructose.^18^ The buffering of the reaction solution was necessary for a stable complex formation between the fructose and the organoboronic acid which is dissolved in the organic phase.^32,33^ A hydrophobic organoboronic acid with a high affinity for the organic phase needs to be applied (e.g., 4-vinylphenylboronic acid^34^). Here, naphthalene-2-boronic acid was used. The organic phase was applied at a phase ratio of one to the aqueous phase. Stripping of the organic phase with nitric acid was necessary to recover fructose and enabled the recycling of the organic phase within the three-step process.^18^ The organic to strip phase ratio was around five for each stripping step. The fructose concentration in the strip stream was thereby increased, making a further utilization possible. With the three-step process, ∼84% of fructose could be extracted from the aqueous into the organic phase. 2-GG and glycerol were extracted by <10 and 13%. The aqueous phase leaving the extraction process had a volume of ∼6.73 L with 2-GG, fructose, and glycerol concentrations of around 25, 4, and 45 g L^–1^. Around 90% of the extracted fructose could be recovered in the strip phase. The strip phase had a volume of ∼3.77 L with 2-GG, fructose, and glycerol concentrations of 2, 31, and 10 g L^–1^.

We observed that the extraction potential of the organic phase decreased after the three-step process. This was not noticed in the small-scale process.^18^ The reason for that could be a leakage of the extraction agent (i.e., organoboronic acid) or a pH drop that impeded the complexation of fructose. In view of the economic and also the ecologic efficiency, recycling of the organic phase is yet of high importance. A pre-activation/regeneration step of the organic phase with buffer before each extraction step could be used to overcome this issue.^35,36^ It was assumed that a high operation stability of the organic phase, without a considerable leakage of the extraction agent, could thus be ensured. Although not shown from this study, Sánchez-Bastardo et al. (2018) report a sugar reactive extraction process that comprised pre-activation of the organic phase, reactive extraction, and acidic stripping. Boron leakage in this process was below 2%.^35^ In another study of reactive extraction of saccharides, the pre-activated organic phase could be constantly recycled 10 times without observable loss of the extraction potential.^34^ We therefore assume that a constantly pre-activated organic phase (and also the pre-activation buffer) could be used minimally for three runs of the three-step process. However, since borate leakage into the aqueous phase during the extraction could not be excluded completely, despite the use of a hydrophobic organoboronic acid and a pre-activated organic phase, an ion-exchange step for borate removal has to be included. Amberlite IRA743 could be used as well-recyclable resin with good selectivity for borate.^37,38^

#### Discontinuous Diafiltration

The aqueous phase (∼6.73 L) from the extraction-stripping process was ultrafiltered and fed into the multistep diafiltration for glycerol removal.^17^ The diafiltration was performed at constant permeate flux (10.0 ± 1.5 kg m^–2^ h^–1^) to counteract the increasing viscosity of the retentate in each cycle. Due to the ongoing removal of glycerol, the required pressure to maintain the set permeate flux dropped from cycle to cycle (1. cycle: 7.8 → 15.3 bar, 5. cycle: 4.0 → 9.5 bar). Five diafiltration cycles were necessary to decrease the glycerol in the retentate to <1 g L^–1^.

The rejection of 2-GG was high, resulting in a 2-GG recovery in the retentate of >85%. The major part of the introduced salts (buffer and pH adjustment) could be also removed in the permeate. Conductivity measurements suggest salt concentrations in the retentate <2 g L^–1^. The retentate had a volume of ∼2.8 L with 2-GG, fructose, and glycerol concentrations of 51, 3, and <1 g L^–1^. The permeate had a volume of ∼25 L with 2-GG, fructose, and glycerol concentrations of 1, <1, and 12 g L^–1^. By the combination of extraction-stripping and diafiltration, 95% of fructose and 99% of glycerol could thus be removed from the 2-GG product solution. The 2-GG purity increased to ∼80 wt %, and the pre-defined thresholds of fructose and glycerol contents of <10 and <2 wt % based on 2-GG were achieved.

### Integrated Process Design

**Figure 2:**
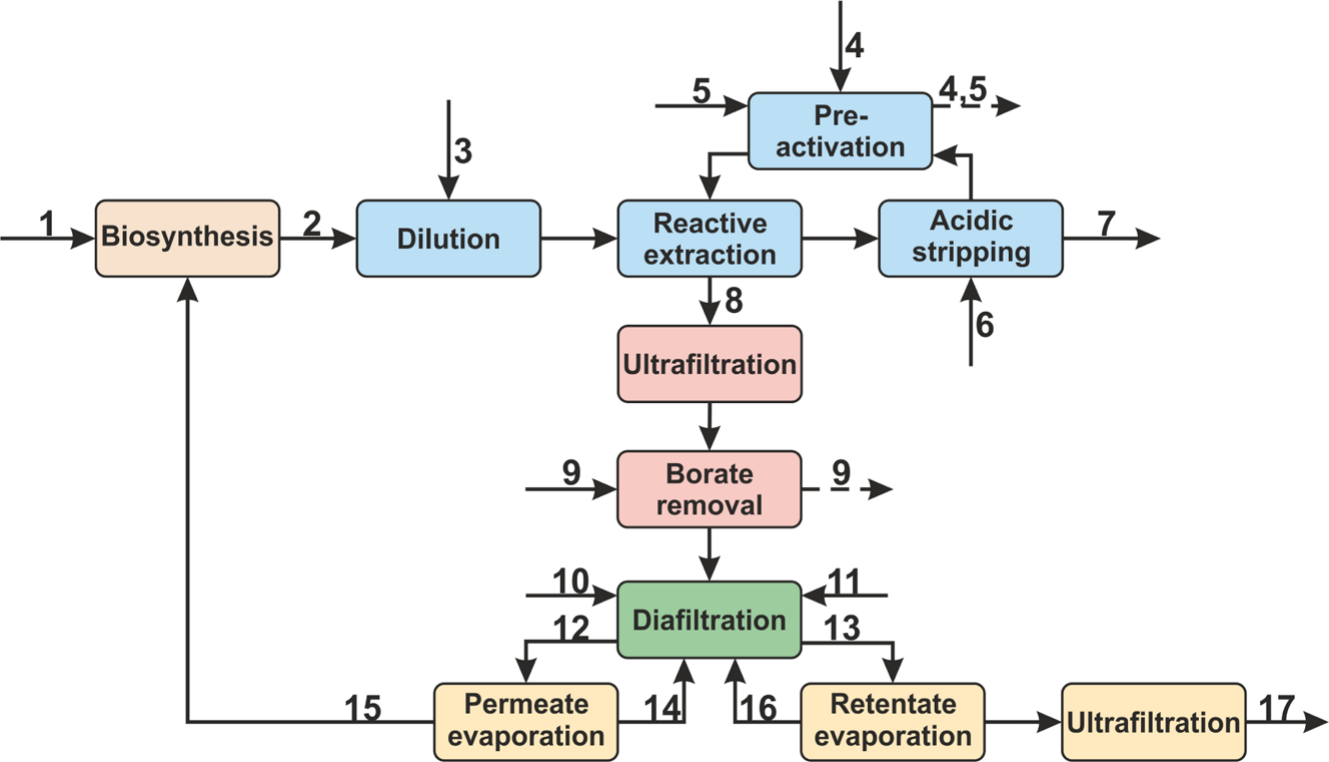
Integrated process design for the production of 2-GG. (1) Substrate feed stream, (2) reaction mixture, (3) dilution buffer, (4) organic phase, (5) pre-activation buffer, (6) strip phase, (7) stripped stream, (8) extracted aqueous phase, (9) regeneration stream resin, (10) dilution stream, (11) diafiltration water, (12) permeate, (13) retentate, (14) recycled water permeate, (15) recycled permeate, (16) recycled water retentate, and (17) product (2-GG). Dashed lines are waste streams.

Based on the evidence described above, an integrated process for 2-GG production evolves as presented in Figure 2. The retentate (13) is the main product stream. For direct application, the 2-GG content in the retentate is too low (∼5 wt %). Therefore, an evaporation step is necessary to remove the surplus of water which can be recycled to the diafiltration step (16). The concentrated retentate is ultrafiltered and gives the final product stream (17) with a 2-GG content of ≥50 wt %. Additionally, two side streams with further utilization potential emerge from the process: the stripped stream (7) with a fructose purity of ∼70 wt % and a recovery of almost 75% and the permeate (12) with a glycerol purity of 86 wt % and a recovery of 83%. The strip stream can be further utilized for HMF production. The permeate needs to be concentrated by evaporation to remove the high amount of diafiltration water. The concentrated permeate (15) can be recycled to the synthesis, while the evaporated water (14) can be reused in diafiltration. The utilization of these two streams is independent of the scale of the process.

#### Fructose Utilization

We evaluated the fructose in the strip stream (∼31 g L^–1^) to produce HMF, a promising platform chemical for the polymer industry.^19,21,29^ HMF can be produced via the hydrothermal dehydration of fructose which is done at up to ∼260 °C using Brønsted acid (pH ≤ 3) as the catalyst. Aqueous solutions with a fructose content of 1–30 wt % are usually used, and the residence times are conventionally <1 h.^39−41^ Such a process is industrially realized by AVA Biochem (Zug, Switzerland). A model stream representing our strip stream was evaluated for HMF production in a proprietary lab-scale process at AVA Biochem. A HMF yield of 27% with a selectivity of 43% was achieved. The fructose conversion was 63%. By way of comparison, in the literature, HMF yields between 25 and up to 70% can be found.^41^ The rather low yield achieved with our stream could be due to side reactions and product degradation. Numerous by-products can evolve during the hydrothermal production of HMF from fructose, including formic and levulinic acid, furfural, or humins.^41^ The high salt loading used might be a relevant factor of enhanced by-product formation in the present case. The salt derives from the addition of NaOH to increase the pH of the strip stream from <1 to ∼2.8, as required for the hydrothermal production of HMF. For desalting without monosaccharide loss, electrodialysis could be used. Galier and Roux-de Balmann (2015) purified a monosaccharide solution by electrodialysis and achieved a demineralization factor of 90% with a monosaccharide loss ≤5%.^42^ Alternatively, the nitric acid concentration of the strip phase could be reduced within the limits of the fructose recovery. Finally, there is the possibility of admixing the fructose from the 2-GG process with other fructose feedstocks in ratios suitable for high HMF selectivity.

#### Glycerol Recycling

The glycerol in the permeate was concentrated to ∼155–160 g L^–1^ and combined with fresh glycerol (160 g L^–1^) at a ratio of ∼2.25:1.00 (v/v) to simulate real process conditions. Glycerol reusability for 2-GG production was analyzed based on sucrose conversion (Figure S1). The recycled glycerol was not useable without pH control. Its elevated pH (∼9) was too high for SucP activity. The high pH is caused by the buffer salts used in the extraction step. The salts are predominantly removed into the permeate during the diafiltration. The pH of the concentrated permeate should be ≤ 8 in order to achieve a sucrose conversion that is comparable with fresh glycerol. In the future, it must be analyzed whether the recycling of the permeate causes a change of the yield and/or the composition of the product solution of the biosynthesis (i.e., unwanted by-products are formed) and whether it has an influence on the downstream processing of the developed process.

### Process Modeling

The process in Figure 2 was modeled with Aspen Plus, assuming an industrial scale production of 10 tons 2-GG solution (50 wt %) per year (annual operation of 7200 h^–1^). Of note, the production scale is an estimated value. The production capacity could be much higher. Both substrates (i.e., glycerol and sucrose) are produced at the million tons scale.^43,44^ Glycerol is available in excess on the market, which is due to the increasing biodiesel industry.^44^ The simulated process implemented in Aspen Plus is presented in Figure S2. The catalyst preparation, the pre-activation of the organic phase, the ultrafiltration steps, and the borate removal were not included in the simulation since it was assumed that they do not cause a major change of the composition of the process streams. However, the required mass and energy inputs of those steps were considered for the process analysis. The mass streams and energy input derived from the Aspen Plus simulations are summarized in Tables S5–S7. The simulation results were used to assess the efficiency and the environmental impact of the developed process and to investigate if green chemistry standards are met. We therefore analyzed the mass of materials used, mass recovered, and mass of waste formed for the developed process. The total mass input was 1475 t a^–1^. Around 80% of the mass could be further utilized (stream 7, 17 in Figure 2) or recycled in the process (stream 14, 15, 16 in Figure 2). The rest was waste, including streams 4 and 5 (assumption: both streams were recycled for three process runs), stream 9, the PAM-I, the ion-exchange resin, the membranes, and the wash water used in the PAM-I production and for membrane cleaning. The *E*-factor (mass waste to mass product) and PMI (total mass to mass product) were thus 28 and 146, respectively.^3,45^ We also considered to omit the recycling of the glycerol, since its concentration is an energy intensive process (≥628 MW h a^–1^). Stream 12 is thus not recovered but treated as a waste. The amount of recovered mass thereby dropped to 18% and the *E*-factor increased to 120. Moreover, to underline the efficiency of the developed process, it was compared to an alternative 2-GG process which comprised no extraction-stripping step but only diafiltration to remove glycerol and fructose. This process is depicted in Figure S3 and described in the Supporting Information. It was calculated that 19–20 diafiltration cycles would be necessary to achieve the glycerol and fructose content in the product solution that meets the product specifications. The resulting 2-GG recovery was <55%. Within these 20 steps, ∼3100 t a^–1^ of diafiltration water would be necessary. This is more than double of the mass input of the here developed process. Due to the simultaneous removal of glycerol and fructose and the high dilution of the permeate, a recovery of these two compounds seems energy- and resource-intensive. Therefore, only the disposal of the permeate could be considered as reasonable. The *E*-factor and PMI were thus 444 and 457, respectively. This shows that the recovery of glycerol and fructose as achieved with the developed process is of high importance to decrease the environmental impact of 2-GG production. Non-benign chemicals are admittedly applied in this process, however, if they are recycled effectively, they are applied in acceptable amounts. Of note, the ratio of the organic phase used in the developed process (recycled for three runs) to the surplus of materials used in the alternative process was <0.04. An overview of the material masses used and recovered as well as of the masses of waste formed in each of the three processes can be found in Table S8.

We further assessed the ecological impact of the proposed 2-GG process by performing an EcoScale analysis.^23^ This analysis assigns penalty points to important process parameters, including yield, price of raw materials, safety of applied chemicals, technical setup, operation conditions, and the downstream process. A perfectly sustainable process exhibits zero penalty points. The EcoScale analysis for the 2-GG process is summarized in Table 1.

**Table 1:** EcoScale Analysis of the Proposed 2-GG Process^a^

parameter		description	penalty points
1.	Yield	2-GG yield (83%) of the reaction was taken	9
2.	Price of reaction components (to obtain 10 mmol of the end product)	∼10 t product with a 2-GG content of 50 wt % is produced annually → 19.7 kmol 2-GG a^–1^ total raw material costs are 566,825 € a^–1^	0
3.	Safety	Hazard compounds:	
		Organic phase of the extraction process^b^	15
		1-Octanol	
		*n*-Heptane (F, N)^c^	
		Napthalene-2-boronic acid	
		Aliquat 336 (N, T)^c^	
		Stripping phase of the stripping process	5
		HNO_3_ (T)^c^	
4.	Technical setup^d^	Pressure equipment	3
		Packed-bed reactor	
		Instruments for controlled addition of chemicals	1
		Pump (constant flow rate)	
5.	Temperature/time	Reaction temperature 40 °C (>1 h)	3
6.	Workup and purification	Dilution (adding solvent)	0
		Extraction-stripping (liquid–liquid extraction)	3
		Ultrafiltration (filtration)	1
		Borate removal (solid phase extraction)	2
		Diafiltration (filtration)	1
		Permeate evaporation (removal of solvent with bp < 150 °C)	0
		Retentate evaporation (removal of solvent with bp < 150 °C)	0
		Ultrafiltration (filtration)	1
Total^e^			44

a A detailed description of the EcoScale can be found in Van Aken et al. (2006).^23^

b It was assumed that the organic phase is one entity. If two compounds shared the same hazard warning symbol, it was only counted as one.

c F, highly flammable; N, dangerous to the environment; and T, toxic.

d Technical setup of the biosynthesis is considered.

e Ranking: <25 excellent, <50 acceptable, and >50 inadequate.

A penalty point score of 44 was determined, which is according to the EcoScale, an acceptable process.^23^ Of note, the final 2-GG purity is 80 wt %, which is suitable for the applications intended but below the designated purity of the EcoScale analysis. The hazardous chemicals used in the extraction-stripping step had the highest contribution. A substitution of those chemicals by more benign ones would thus increase the EcoScale score. Moreover, an enhancement of the yield would be also beneficial for the EcoScale score.

### Techno-Economic Assessment

The TEA is the basis for each tangible process development and represents a powerful process analysis tool. It reveals critical process steps and economic limitations of the process. As a central point, the profitability of the process is assessed, which is an important decision criterion of whether realization of the process at the production scale is reasonable. We performed a TEA for the process depicted in Figure 2 and used the Aspen Plus simulations for it. Results utilized for the TEA are summarized in Tables S5–S7.

#### Fixed Capital Investment

The TEC are the basis for estimating the TCI. The TEC were estimated with Aspen Plus. The equipment was sized according to the calculated mass streams. A detailed listing of the required equipment, including their sizing and their costs, is summarized in Table S9. The TEC ran up to ∼502,000 €. The main contributors were diafiltration, the permeate concentration, and the extraction-stripping step with 32, 31, and 19% of the TEC. The diafiltration and the concentration of the permeate required highly engineered and expensive equipment (i.e., cross flow nanofiltration device and thin-film evaporator). In the extraction-stripping step, no expensive equipment is used, yet, due to the high number of mixing tanks, agitators, and separation tanks needed, the equipment costs were still high. For each step (i.e., extraction, stripping and organic phase pre-activation), three mixing tanks, agitators, and separation tanks were required. The resulting TCI was ∼2.82 million €. A summary of the TCI can be found in Table S10.

#### Operating Costs

**Figure 3:**
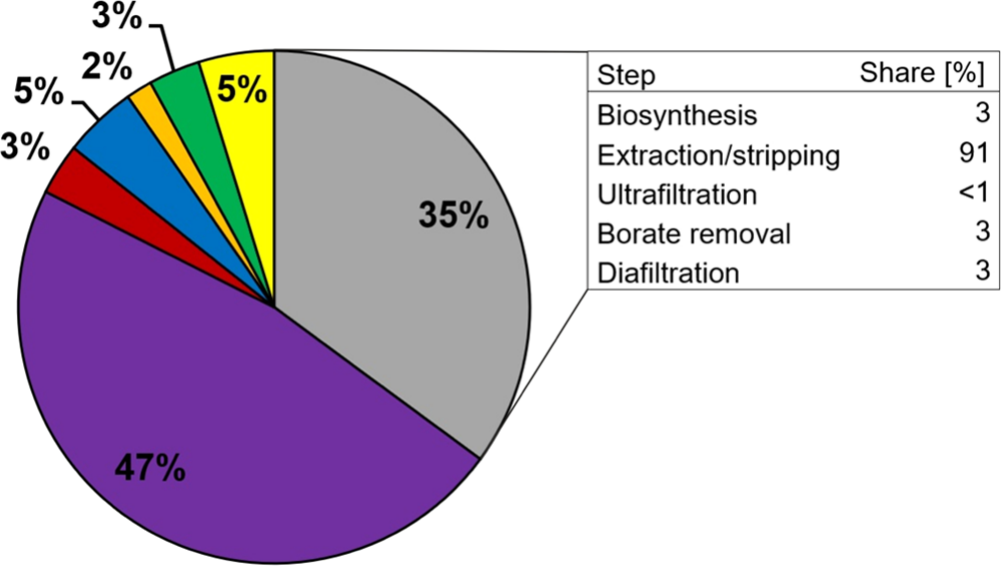
Distribution of the operation costs, including costs for labor (purple), waste treatment (red), utilities (blue), insurance (orange), maintenance (green), overhead (yellow), and raw materials (grey), including the share of the process steps to the raw material costs.

The OC added up to ∼1.62 million €. The distribution of the OC is depicted in Figure 3 and listed in Table S11. The labor costs represented the highest share of the OC. The required staff to run the process is listed in Table S3. The raw material costs had the second highest impact. As shown in Figure 3, the main contributor was the extraction-stripping step with 91%. The organoboronic acid and the phase transfer catalyst accounted for ∼71% of the total raw material costs. This shows that a recycling of the organic phase for several times is key to keep the raw material costs at a reasonable level. Of note, we assumed that the organic phase could be used for three runs of the three-step extraction-stripping process, as mentioned above. Every further recycling run would considerably reduce the OC and would be also ecologically beneficial. The utility costs had only a low impact (∼5%). A deeper analysis thereof provided important information with regard to the design of the process line. The utility costs required for the concentration step of the permeate stream (i.e., evaporation, condensation, and cooling) to enable the recycling of the unconverted glycerol (∼11,100 kg a^–1^) were determined to be ≥50,000 € a^–1^. By way of comparison, by the direct disposal of the permeate, materials with a value of ∼7500 € are lost and additional costs for waste treatment (i.e., the permeate is assumed to be water waste) of ∼23,200 € arise. Economically, it thus does not make sense to concentrate and recycle the permeate. The high equipment costs for this process step (∼200,000 €) further emphasize a more economic approach of using fresh glycerol instead of concentrating the permeate for glycerol recycling. Yet, the developed process strives strongly to minimize waste formation as presented above. Utilization of the permeate stream is therefore favored.

#### Assessment of the Profitability

The process costs (TCI and OC) were compared with the potential revenues of the production process to examine its profitability. To consider the time value of money, a DCFA was performed. As an economic index, the MSP for 2-GG was calculated.^26,27,29,30^ In the literature, no accurate selling price for 2-GG was found. According to the literature, the price for high-value products (e.g., pharmaceuticals and cosmetics) is in the range of 100 to 1000 US$ kg^–1^.^7,46^ We retained the price in the lower limit and assumed a price between 100 and 300 € kg^–1^. The main revenue is generated by the sale of the 2-GG solution (∼10 t a^–1^, stream 17, see Figure 2). The strip stream (stream 7) of the extraction process (∼155 t a^–1^) could be also divested. The only exploitable compound of this stream is fructose, which is present at a concentration of ∼27 g kg^–1^. The commercial fructose syrup with purities up to 90% usually costs between 500 and 1000 € kg^–1^. The strip stream is also contaminated with other compounds therefore a selling price of 0.01 € kg^–1^ was assumed, resulting in a revenue in the first year of only ∼1550 €. For the investigated production scale, the fructose recovery thus does not add economic value to the process. However, it could become relevant for a larger production scale. Moreover, the fructose recovery increases the ecologic efficiency since waste formation is avoided by further utilization. An additional return of the process is the recovered glycerol stream (stream 15) and the water streams (stream 14, 16) of the evaporation steps. The determined MSP for 1 kg 2-GG solution was 189 €. A detailed overview of the calculations of the DCFA can be found in Table S12.

#### Sensitivity Analysis

**Figure 4:**
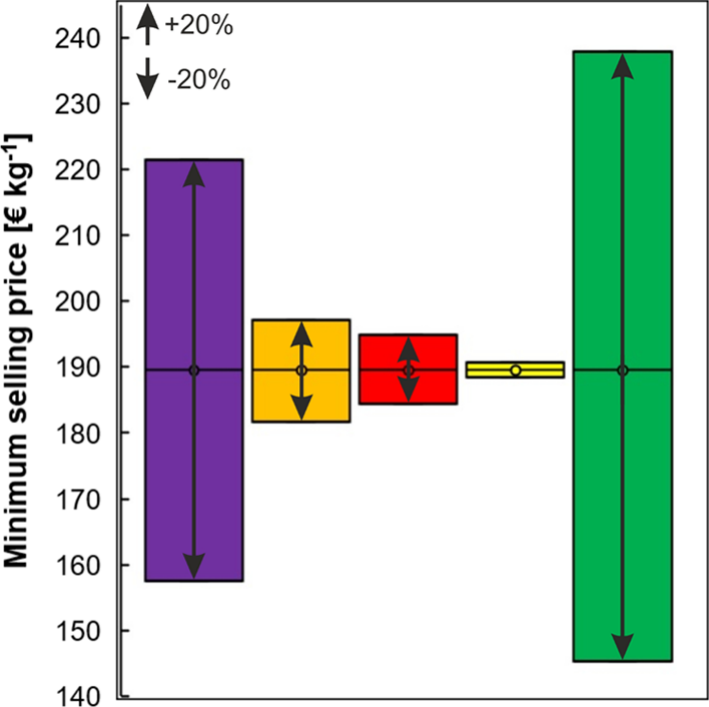
Sensitivity analysis including the variation of the operating costs (purple), TECs (orange), discount rate (red), income tax (yellow), and the worst/best case scenario where the four quantities were varied simultaneously.

The TEA is based on some assumptions which causes an uncertainty of the calculated values. A sensitivity analysis is therefore important to check if the process is still profitable if economic and process parameters vary.^26,27,29^ The influence of OC, TEC, discount rate, and income tax on the MSP was analyzed by varying these quantities in a range of −20 and +20%.^27,29^ The MSP was also calculated for the worst/best case scenario, where all four quantities were simultaneously varied by either −20 or +20%. The results of the sensitivity analysis are summarized in Figure 4. The MSP was within the price range mentioned above for all tested scenarios. The OC had the highest influence, causing a change of the MSP by ±17%. The other three quantities had a much smaller effect on the MSP (less than ±5%). In the worst/best case scenario, the MSP varied by around ±25%. Additionally, to these four main quantities, we also varied the number of recycling runs of the organic phase, since it was observed that it had a considerable impact on the operating costs as shown above. If the number of recycling runs of the organic phase could be increased to five or seven, the MSP decreased to 167 and 157 € kg^–1^.

## Conclusions

An integrated process for the production of 2-GG for functional ingredient use was shown for individual unit operations performed at an aligned scale. The process was assessed for ecological impact and analyzed by a TEA. Individual unit operations developed in earlier research (continuous bioconversion in a packed-bed reactor containing immobilized whole cells; reactive extraction-acidic stripping; and discontinuous diafiltration) were interconnected to establish a holistic process. The production process yielded the aqueous solution of 2-GG in the required purity (80 wt %) and enabled a recovery of >70% of the unconverted glycerol as well as of the by-product fructose. The glycerol could be recycled for re-use, and the fructose was a potential feedstock for HMF production. Due to the recovery of high amounts of the input mass, waste formation was minimized. EcoScale analysis revealed a penalty point score of 44, suggesting an acceptable process from sustainability and eco-credential points of view. The TEA revealed opportunities and limitations of the process. The reactive extraction-acidic stripping step was crucial. A stable organic phase is required that can be effectively recycled for several extraction cycles. The economic efficiency as well as the sustainability of the here developed process would thus be increased. Overall, the DCFA showed the profitability of the 2-GG process. MSPs (<240 € kg^–1^) reasonable for this product category were obtained. This study provides important findings that could facilitate the implementation of a fully integrated 2-GG process at the industrial scale. Moreover, it shows that the implementation of a lab-scale process, monitoring of relevant process data, process simulation, and a TEA are parts that should be closely connected during the development of new bio-processes.

## Supporting Information Available

Equations of technical and economic parameters; performance of glycerol recycling; flow sheet of the process simulated in Aspen Plus; design specification of the Aspen Plus process; material price list; staff and salary list; parameters of the discounted cash flow assessment; mass streams and utilities required for an industrial process; alternative 2-GG process; required process equipment; total capital investment and operation costs; and discounted cash flow calculations (PDF)

## Notes

Data obtained in the current study are available from the corresponding author (B.N.) upon reasonable request.

## Abbreviations

2-GG: 2-*O*-α-d-glucosyl-glycerol
DCFA: discounted cash flow assessment
*E*-factor: environmental factor
FCI: fixed capital investment
FOC: fixed operating costs
HMF: 5-(hydroxymethyl)furfural
MSP: minimum selling price
OC: operating costs
OCC: other capital costs
PAM: polyacrylamide
PAM-I: polyacrylamide-immobilizate
SucP: sucrose phosphorylase
TCI: total capital investment
TDC: total direct costs
TEC: total equipment costs
TIC: total indirect costs
VOC: variable operating costs
WC: working capital
