# Supplementary material for: Biocatalytic Production of 2-α-d-Glucosyl-glycerol for Functional Ingredient Use: Integrated Process Design and Techno-Economic Assessment
Source: ACS Sustain Chem Eng. 2022 Jan 11;10(3):1246–55. doi: 10.1021/acssuschemeng.1c07210 (PMC8790807; doi:10.1021/acssuschemeng.1c07210)
Supplement: Supplementary file 1 — sc1c07210_si_001.pdf [file sc1c07210_si_001.pdf]

Supporting Information

Biocatalytic production of 2-α-D-glucosyl-glycerol for functional ingredient use: Integrated process design and techno-economic assessment

Andreas KRUSCHITZ[†,‡] *and Bernd NIDETZKY*[†,‡,*]

†Austrian Centre of Industrial Biotechnology (acib), Krenngasse 37, 8010 Graz. Austria

‡Institute of Biotechnology and Biochemical Engineering, Graz University of Technology, NAWI Graz, Petersgasse 12, 8010 Graz. Austria

*Corresponding author, E-mail: bernd.nidetzky@tugraz.at; phone: +433168738400; fax: +433168738434

24 Pages

Technical and economic parameters used

3 Figures (Figure S1 – S3)

12 Tables (Table S1 – S12)

Technical parameters

The space velocity (SV), the 2-GG selectivity (S_{2-GG}, mole basis), the 2-GG regioselectivity (RS_{2-GG}) and the purity of compound i (Pu_i) were defined as:

$$SV = \frac{\dot{v}}{V} = \frac{1}{\tau} \quad \text{S1}$$

$$S_{2-GG} = \frac{c_{2-GG}}{c_{Fructose}} \quad \text{S2}$$

$$RS_{2-GG} = \frac{c_{2-GG}}{c_{2-GG} + c_{1-GG}} \quad \text{S3}$$

$$Pu_i = \frac{c_i}{c_{Fructose} + c_{1-GG} + c_{2-GG} + c_{Glycerol} + c_{Glucose} + c_{Sucrose}} \quad \text{S4}$$

$\dot{v}$ was the flow rate, V was the reactor volume, τ was the mean residence time, c was the concentration. The 2-GG selectivity (S_{2-GG}) was calculated based on the molar amount of fructose formed, which represents the highest amount of 2-GG that can be theoretically formed. SucP also catalyzes the formation of glucose (due to sucrose hydrolysis, Scheme 1) and of the isomer 1-α-D-glucosyl-glycerol.[1,2] In total, the 2-GG yield is thus lowered compared to fructose.

Economic parameters

The discounted cash flow (DCF) and the net present value (NPV) were defined as:

$$DCF = \sum_{i=1}^{n} \frac{CF_i}{(1+r)^i} \quad \text{S5}$$

$$NPV = DCF - TCI \quad \text{S6}$$

CF was the net cash flow, r was the discount rate, TCI was the total capital investment which is the sum of the fixed capital investment and the working capital. The index i represents the investigated years.

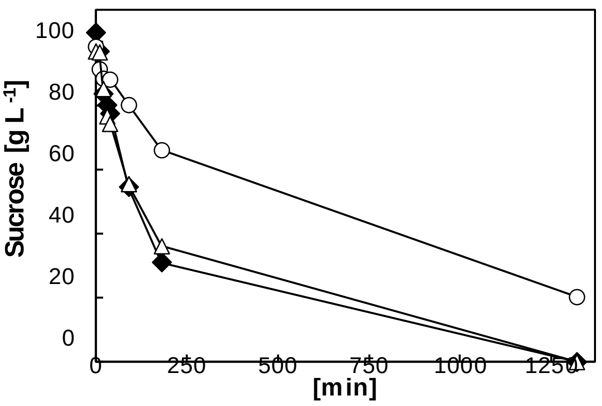

Time [min]	♦	○	Δ
0	102.8	98.5	96.8
10	97.0	91.2	96.7
20	83.6	88.5	85.2
30	80.3	-	76.8
40	77.6	88.2	74.3
90	54.6	80.2	55.4
180	31.0	66.2	36.1
1320	0.0	20.3	0.0

Figure S1. Sucrose conversion with fresh glycerol (pH~7.2) (♦), recycled glycerol (pH~9.0) (○) and recycled glycerol with adjusted pH (pH~8.0) (Δ). The starting glycerol concentration was 156±5 g L^{-1} and PAM-I (0.25-2.0 mm) was used as biocatalyst. In the table next to the graph the corresponding concentrations [g L^{-1}] are listed.

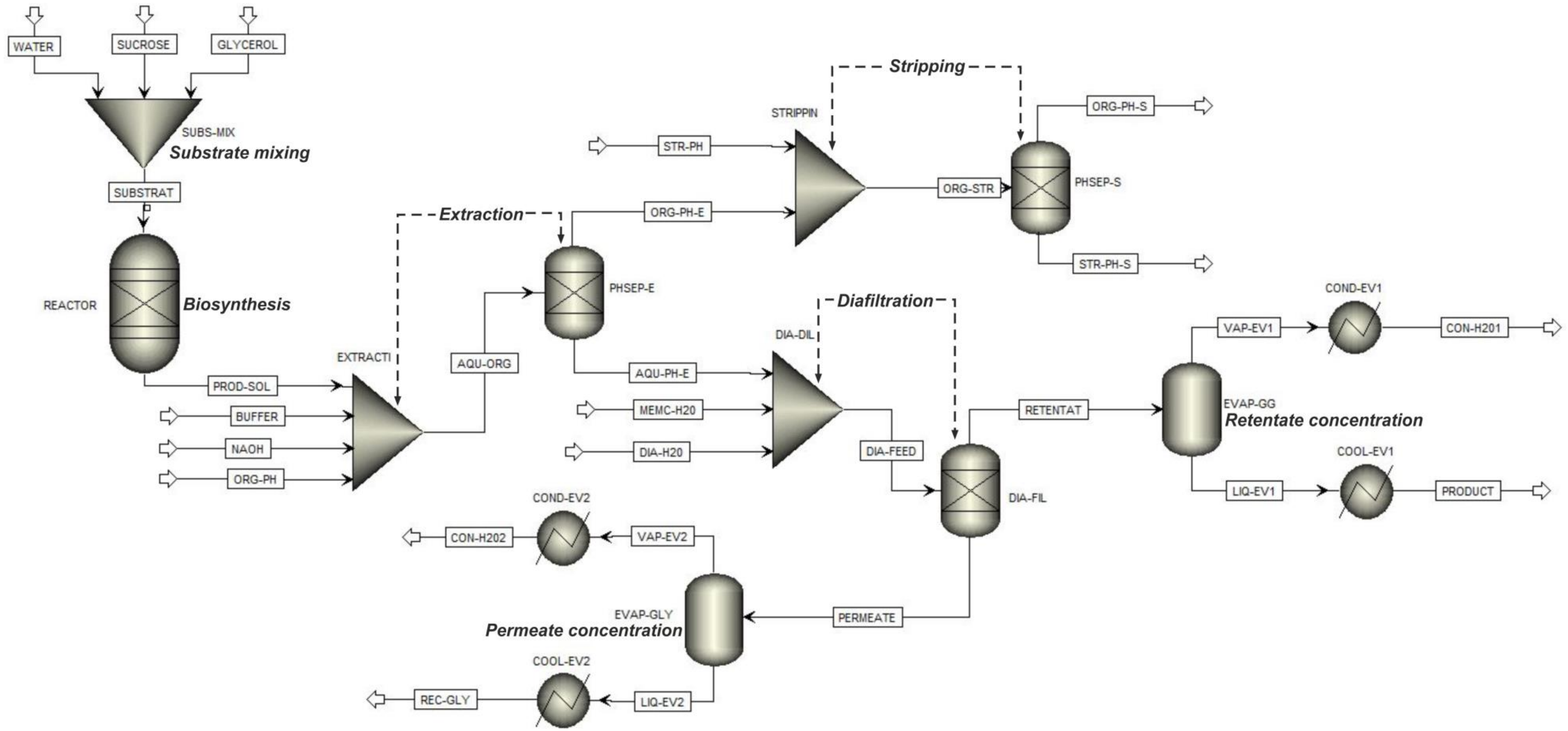

Figure S2. 2-GG process design implemented in Aspen Plus®.

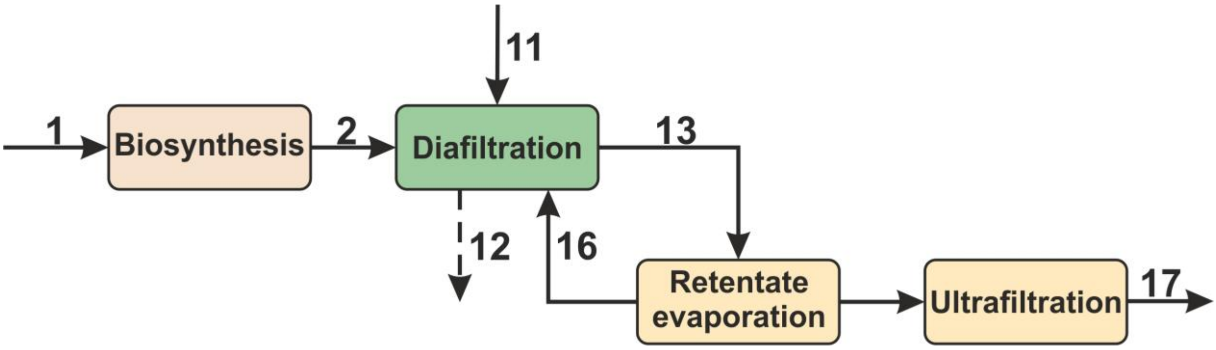

Figure S3. Alternative process design for the production of 2-GG. 1 Feed stream, 2 Reaction mixture, 11 Diafiltration water, 12 Permeate, 13 Retentate, 16 Recycled water retentate, 17 Product (2-GG). The dashed line represents a waste stream.

The process depicted in Figure S3 comprised continuous biosynthesis in a packed-bed reactor filled with PAM-I particles as already described for the process depicted in the main text Figure 2. The effluent (stream 2, ~95 t a^{-1}) had the same composition as stream 2 in Figure 2. It is directly processed by discontinuous diafiltration. Glycerol and fructose are thereby removed in the permeate (12) and 2-GG retained in the retentate (13). To increase the 2-GG content in the retentate, it is concentrated by evaporation. The surplus of water (16) can be recycled back to the diafiltration. In a last step the concentrated retentate is ultrafiltered to give the final product solution (17). The diafiltration process was analyzed based on a mass balance model that was presented previously.[14] It was assumed that the solute rejection was constant with mean rejection coefficients of glycerol, fructose and 2-GG of around 2, 79 and 95%. The concentration factor was assumed to be around 2.75.

Table S1. Design specifications of the process steps modeled in Aspen Plus®

Process step	Aspen unit	Conditions	Specification	Utility
SUBS-MIX	Mixer	1.013 bar		
REACTOR	RYield	40 °C<br>1.013 bar	Component yields (mass basis):<br>Fructose: 0.05754<br>1-GG: 0.00930<br>2-GG: 0.06795<br>Glucose: 0.00275<br>Glycerol: 0.13471<br>Sucrose: 0.00305<br>Water: 0.72470	
EXTRACTI	Mixer	1.013 bar		
PHSEP-E	Sep		Split fraction (outlet stream: AQU-PH-E):<br>Fructose: 0.159<br>1-GG: 0.910[a]<br>2-GG: 0.910<br>Glucose: 0.800[b]<br>Glycerol: 0.874<br>Na_2CO_3: 1.000<br>$NaHCO_3$: 1.000<br>NaOH: 1.000<br>Sucrose: 1.000[c]<br>Water: 1.0000<br>Rest: 0.0	
STRIPPIN	Mixer	1.013 bar		
PHSEP-S	Sep		Split fraction (outlet stream: STR-PH-S):<br>Fructose: 0.894<br>1-GG: 0.450[a]<br>2-GG: 0.450<br>Glucose: 0.950[b]<br>Glycerol: 0.869<br>HNO_3: 1.000<br>Water: 1.000<br>Rest: 0.0	
DIA-DIL	Mixer	1.013 bar		
DIA-FIL	Sep		Split fraction (outlet stream: RETENTAT):<br>Fructose: 0.303<br>1-GG: 0.861[a]<br>2-GG: 0.861<br>Glucose: 0.303[b]<br>Glycerol: 0.006<br>Na_2CO_3: 0.070[d]<br>$NaHCO_3$: 0.070[d]<br>NaOH: 0.070[d]<br>Sucrose: 1.000[c]<br>Water: 0.100<br>Rest: 0.0	
EVAP-GG	Flash2	1.013 bar	Design specification:<br>Mass-Frac of 2-GG in LIQ-EV1 is 0.5<br>Manipulated variable:<br>Temperature of block EVAP-GG (100-120 °C)	Electricity
COND-EV1	Heater	23 °C<br>1.013 bar	Cooling water temperature: 10 → 30 °C	Cooling water
COOL-EV1	Heater	23 °C<br>1.013 bar	Cooling water temperature: 10 → 30 °C	Cooling water
EVAP-GLY	Flash2	1.013 bar	Design specification:<br>Mass-Frac of glycerol in LIQ-EV2 is 0.168<br>Manipulated variable:<br>Temperature of block EVAP-GLY (100-110 °C)	Electricity
COND-EV2	Heater	23 °C<br>1.013 bar	Cooling water temperature: 10 → 30 °C	Cooling water
COOL-EV2	Heater	23 °C<br>1.013 bar	Cooling water temperature: 10 → 30 °C	Cooling water

[a] 1-GG: It is assumed that 1-GG behaves similarly as 2-GG in the extraction/stripping and diafiltration process, therefore the same split fractions were used.

[b] Glucose: Glucose extraction is hardly quantifiable since glucose is present at low concentrations. It was assumed that 20% of glucose is extracted and 95% is then stripped. In the diafiltration it is assumed that glucose has a similar retention coefficient as fructose and thus the same split fraction.
[c] Sucrose: It was assumed that sucrose is neither removed by extraction/stripping nor by diafiltration.
[d] Ions: It was assumed that more than 93% of the ions are removed by diafiltration.

Table S2. Prices used for the techno-economic assessment

Material	Price	Reference
Aliquat 336	40 € kg^{-1}	Alibaba.com
Anion exchanger (comparable to Amberlite IRA743)	17 € L^{-1}	Alibaba.com
Cooling water	0.135 € t^{-1}	[3–5]
Electricity	0.074 € kWh^{-1}	[3–8]
Glycerol	0.520 € kg^{-1}	[9]
n-Heptane	1.800 € kg^{-1}	Alibaba.com
H_2SO_4	0.213 € kg^{-1}	[6,9]
HCl (36%)	0.250 € kg^{-1}	Made-in-china.com
HNO3 (65%)	0.290 € kg^{-1}	Made-in-china.com
Immobilizate PAM-I	50 € kg^{-1}	Calculated based on chemicals used
Membrane	231 € m^{-2}	[7]
Na_2CO_3	0.200 € kg^{-1}	Alibaba.com, made-in-china.com
$NaHCO_3$	0.230 € kg^{-1}	Alibaba.com, made-in-china.com
NaOH	0.350 € kg^{-1}	[4,9]
1-Octanol	1.700 € kg^{-1}	Alibaba.com, made-in-china.com
Organoboronic acid (Naphthalene-2-boronic acid)	110 € kg^{-1}	Alibaba.com
Sucrose	0.300 € kg^{-1}	[9–11]
Water waste (treatment/disposal)	25 € t^{-1}	[3]
Aqueous waste (treatment/disposal)	151 € t^{-1}	[3]
Hazard waste (treatment/disposal)	317 € t^{-1}	[3,4]
Solid waste (treatment/disposal)	16 € t^{-1}	[6]
Water	0.750 € t^{-1}	[4,5,9,12]

Table S3. Required staff and their associated salaries (based on the Austrian wage agreements for the chemical industry)

Position	Quantity	Salary category	Salary [€ $month^{-1}$]
Plant supervisor	1	V	4,527.78
Plant technician	1	IV	3,389.68
Lab technician	1	III	2,696.88
Shift operator	8	II	2,222.50
Secretary	1	I	1,967.30

[a] It is assumed that the production is carried out in a two-shift (10 h each) and three-shift (8 h each) operation with up to two operators per shift.

Table S4. Parameters used for the discounted cash flow assessment

Annual production capacity (2-GG)	~10 t
Plant life	25 a[4,5,9]
Annual operation	7,200 h
Discount rate	10%[5,9,12]
Income tax	30%[7]
Depreciation	5 years (linear)[5]
Start-up time	0 a[5,9]
Plant salvage value	0 €[5,9,12]

Table S5. Mass streams calculated by the Aspen Plus® simulation

Stream	WATER	SUCROSE	GLYCEROL	SUBSTRAT	PROD-SOL	BUFFER	NAOH	ORG-PH
From	Feed stream	Feed stream	Feed stream	SUBS-MIX	REACTOR	Feed stream	Feed stream	Feed stream
To	SUBS-MIX	SUBS-MIX	SUBS-MIX	REACTOR	EXTRACTI	EXTRACTI	EXTRACTI	EXTRACTI
Mass flow [kg h^{-1}]	9.590	1.442	2.167	13.199	13.199	22.500	0.694	27.720
2-GG [kg h^{-1}]	0.000	0.000	0.000	0.000	0.897	0.000	0.000	0.000
Fructose [kg h^{-1}]	0.000	0.000	0.000	0.000	0.759	0.000	0.000	0.000
Glycerol [kg h^{-1}]	0.000	0.000	2.167	2.167	1.778	0.000	0.000	0.000
Sucrose [kg h^{-1}]	0.000	1.442	0.000	1.442	0.040	0.000	0.000	0.000
1-GG [kg h^{-1}]	0.000	0.000	0.000	0.000	0.123	0.000	0.000	0.000
Glucose [kg h^{-1}]	0.000	0.000	0.000	0.000	0.036	0.000	0.000	0.000
Water [kg h^{-1}]	9.590	0.000	0.000	9.590	9.565	21.814	0.482	0.000
n-Heptane [kg h^{-1}]	0.000	0.000	0.000	0.000	0.000	0.000	0.000	4.222
1-Octanol [kg h^{-1}]	0.000	0.000	0.000	0.000	0.000	0.000	0.000	20.324
HNO_3 [kg h^{-1}]	0.000	0.000	0.000	0.000	0.000	0.000	0.000	0.000
$NaHCO_3$ [kg h^{-1}]	0.000	0.000	0.000	0.000	0.000	0.071	0.000	0.000
Na_2CO_3 [kg h^{-1}]	0.000	0.000	0.000	0.000	0.000	0.615	0.000	0.000
Org.bor.ac. [kg h^{-1}]	0.000	0.000	0.000	0.000	0.000	0.000	0.000	0.596
Aliquat 336 [kg h^{-1}]	0.000	0.000	0.000	0.000	0.000	0.000	0.000	2.578
NaOH [kg h^{-1}]	0.000	0.000	0.000	0.000	0.000	0.000	0.212	0.000
Stream	AQU-ORG	ORG-PH-E	STR-PH	ORG-STR	ORG-PH-S	STR-PH-S	AQU-PH-E	MEMC-H2O
From	EXTRACTI	PHSEP-E	Feed stream	STRIPPIN	PHSEP-S	PHSEP-S	PHSEP-E	Feed stream
To	PHSEP-E	STRIPPIN	STRIPPIN	PHSEP-S	3x recycled → hazard waste	HMF production	DIA-DIL	DIA-DIL
Mass flow [kg h^{-1}]	64.113	28.682	20.710	49.392	27.868	21.524	35.431	5.160
2-GG [kg h^{-1}]	0.897	0.081	0.000	0.081	0.044	0.036	0.816	0.000
Fructose [kg h^{-1}]	0.759	0.639	0.000	0.639	0.068	0.571	0.121	0.000
Glycerol [kg h^{-1}]	1.778	0.224	0.000	0.224	0.029	0.195	1.554	0.000
Sucrose [kg h^{-1}]	0.040	0.000	0.000	0.000	0.000	0.000	0.040	0.000
1-GG [kg h^{-1}]	0.123	0.011	0.000	0.011	0.006	0.005	0.112	0.000
Glucose [kg h^{-1}]	0.036	0.007	0.000	0.007	0.000	0.007	0.029	0.000
Water [kg h^{-1}]	31.862	0.000	18.929	18.929	0.000	18.929	31.862	5.160
n-Heptane [kg h^{-1}]	4.222	4.222	0.000	4.222	4.222	0.000	0.000	0.000

1-Octanol [kg h^{-1}]	20.324	20.324	0.000	20.324	20.324	0.000	0.000	0.000
HNO_3 [kg h^{-1}]	0.000	0.000	1.781	1.781	0.000	1.781	0.000	0.000
$NAHCO_3$ [kg h^{-1}]	0.071	0.000	0.000	0.000	0.000	0.000	0.071	0.000
NA_2CO_3 [kg h^{-1}]	0.615	0.000	0.000	0.000	0.000	0.000	0.615	0.000
Org.bor.ac. [kg h^{-1}]	0.596	0.596	0.000	0.596	0.596	0.000	0.000	0.000
Aliquat 336 [kg h^{-1}]	2.578	2.578	0.000	2.578	2.578	0.000	0.000	0.000
NaOH [kg h^{-1}]	0.212	0.000	0.000	0.000	0.000	0.000	0.212	0.000
Stream	DIA-H2O	DIA-FEED	RETENTAT	LIQ-EV1	PRODUCT	VAP-EV1	CON-H2O1	PERMEAT
From	Feed stream	DIA-DIL	DIA-FIL	EVAP-GG	COOL-EV1	EVAP-GG	COND-EV1	DIA-FIL
To	DIA-DIL	DIA-FEED	EVAP-GG	COOL-EV1	Main product	COND-EV1	Reused for diafiltration	EVAP-GLY
Mass flow [kg h^{-1}]	103.470	144.061	15.006	1.404	1.404	13.602	13.602	129.056
2-GG [kg h^{-1}]	0.000	0.816	0.703	0.703	0.703	0.000	0.000	0.113
Fructose [kg h^{-1}]	0.000	0.121	0.037	0.036	0.036	0.001	0.001	0.084
Glycerol [kg h^{-1}]	0.000	1.554	0.009	0.009	0.009	0.000	0.000	1.545
Sucrose [kg h^{-1}]	0.000	0.040	0.040	0.040	0.040	0.000	0.000	0.000
1-GG [kg h^{-1}]	0.000	0.112	0.096	0.096	0.096	0.000	0.000	0.016
Glucose [kg h^{-1}]	0.000	0.029	0.009	0.009	0.009	0.000	0.000	0.020
Water [kg h^{-1}]	103.470	140.492	14.049	0.448	0.448	13.601	13.601	126.442
n-Heptane [kg h^{-1}]	0.000	0.000	0.000	0.000	0.000	0.000	0.000	0.000
1-Octanol [kg h^{-1}]	0.000	0.000	0.000	0.000	0.000	0.000	0.000	0.000
HNO_3 [kg h^{-1}]	0.000	0.000	0.000	0.000	0.000	0.000	0.000	0.000
$NAHCO_3$ [kg h^{-1}]	0.000	0.071	0.005	0.005	0.005	0.000	0.000	0.066
NA_2CO_3 [kg h^{-1}]	0.000	0.615	0.043	0.043	0.043	0.000	0.000	0.572
Org.bor.ac. [kg h^{-1}]	0.000	0.000	0.000	0.000	0.000	0.000	0.000	0.000
Aliquat 336 [kg h^{-1}]	0.000	0.000	0.000	0.000	0.000	0.000	0.000	0.000
NaOH [kg h^{-1}]	0.000	0.212	0.015	0.015	0.015	0.000	0.000	0.197
Stream	LIQ-EV2	REC-GLY	VAP-EV2	CON-H2O2				
From	EVAP-GLY	COOL-EV2	EVAP-GLY	COND-EV2				
To	COOL-EV2	Reused for the biosynthesis	COND-EV2	Reused for diafiltration				
Mass flow [kg h^{-1}]	9.169	9.169	119.887	119.887				
2-GG [kg h^{-1}]	0.113	0.113	0.000	0.000				
Fructose [kg h^{-1}]	0.083	0.083	0.002	0.002				

Glycerol [kg h^{-1}]	1.541	1.541	0.004	0.004					
Sucrose [kg h^{-1}]	0.000	0.000	0.000	0.000					
1-GG [kg h^{-1}]	0.016	0.016	0.000	0.000					
Glucose [kg h^{-1}]	0.020	0.020	0.000	0.000					
Water [kg h^{-1}]	6.561	6.561	119.882	119.882					
n-Heptane [kg h^{-1}]	0.000	0.000	0.000	0.000					
1-Octanol [kg h^{-1}]	0.000	0.000	0.000	0.000					
HNO_3 [kg h^{-1}]	0.000	0.000	0.000	0.000					
$NaHCO_3$ [kg h^{-1}]	0.066	0.066	0.000	0.000					
Na_2CO_3 [kg h^{-1}]	0.572	0.572	0.000	0.000					
Org.bor.ac. [kg h^{-1}]	0.000	0.000	0.000	0.000					
Aliquat 336 [kg h^{-1}]	0.000	0.000	0.000	0.000					
NaOH [kg h^{-1}]	0.197	0.197	0.000	0.000					

Table S6. Additional materials, which were not listed in the mass stream table (Table S5) or could not be distracted thereof, and their required quantity and waste disposal strategy

Specification (process step)	Material	Quantity	Waste disposal
Immobilizate (biosynthesis)	PAM-I[a]	150 kg a^{-1}	Solid waste
	Water (washing)	1,000 kg a^{-1}	Aqueous waste
Pre-activation phase (extraction/stripping)	Na_2CO_3	2,250 kg a^{-1}	Aqueous waste
	$NaHCO_3$	260 kg a^{-1}	
	Water	82,000 kg a^{-1}	
Borate removal	Resin[b] (10x regenerated)	1,000 L a^{-1}	Solid waste
	water	115,000 kg a^{-1}	Aqueous waste
	H_2SO_4	3,180 kg a^{-1}	
	NaOH	417 kg a^{-1}	
Ultrafiltration (diafiltration)	Membrane[c]	5 m^2 a^{-1}	Solid waste
	Water (washing)	10,000 kg a^{-1}	Water waste
Nanofiltration (diafiltration)	Membrane[c]	61.54 m^2 a^{-1}	Solid waste
	HCl (pH adjustment permeate)	500 kg a^{-1}	

[a]Assumption 1: The PAM-I provides a stable activity for 40 days[13]

[b]Assumption 2: The Amberlite IRA743 resin has a density of 0.7 kg L^{-1}

[c]Assumption 3: One m^2 membrane has a mass of one kg

Table S7. Utilities required per process step

Process step	Unit	Quantity [kWh a^{-1}][a] or [kg a^{-1}][b]	Source
Biocatalyst preparation	Agitation	3,600[a]	calculated/estimated
Biosynthesis	Agitation	7,200[a]	calculated/estimated
	Heating	7,200[a]	calculated/estimated
	Pumping	14,400[a]	calculated/estimated
Extraction/stripping	Agitation	21,600[a]	calculated/estimated
	Agitation	21,600[a]	calculated/estimated
	Agitation	21,600[a]	calculated/estimated
Borate removal	Pumping	14,400[a]	calculated/estimated
Ultrafiltration	Pumping	14,400[a]	calculated/estimated
Nanofiltration	Agitation	14,400[a]	calculated/estimated
	Pumping	72,000[a]	calculated/estimated
Evaporation 1 (2-GG)	Heating	117,889[a]	Aspen Plus®
Condensation 1	Cooling	3,041,906[b]	Aspen Plus®
Cooler 1	Cooling	72,000[b]	calculated/estimated
Evaporation 2 (glycerol)	Heating	627,926[a]	Aspen Plus®
Condensation 2	Cooling	26,764,128[b]	Aspen Plus®
Cooler 2	Cooling	468,000[b]	calculated/estimated

Table S8. Input mass streams, mass recovered and mass waste of the three investigated processes. The processes are depicted in Figure 2 and Figure S3. The unit of the mass streams is tons per year.

Stream	Process w. permeate recycling	Process w.o. permeate recycling	Alternative process
	Input		
PAM-I	0.15	0.15	0.15
Wash water PAM-I	1.00	1.00	1.00
Stream 1	95.03	95.03	95.03
Stream 3	162.00	162.00	0.00
Stream 4	66.53	66.53	0.00
NaOH	5.00	5.00	0.00
Stream 5	84.51	84.51	0.00
Stream 6	149.11	149.11	0.00
Stream 9 + resin	119.30	119.30	0.00
Stream 10 & 11	782.14	782.14	3100.00
Wash water UF	10.00	10.00	5.00
Membranes	0.07	0.07	0.25
HCl (pH adjustment)	0.50	0.00	0.00
	Recovery		
Stream 17	10.11	10.11	7.00
Stream 7	154.97	154.97	0.00
Stream 14	863.19	0.00	0.00
Stream 15 + HCl	66.52	0.00	0.00
Stream 16	97.93	97.93	88.00
	Waste		
PAM-I	0.15	0.15	0.15
Wash water PAM-I	1.00	1.00	1.00
Stream 4 (waste)	66.88	66.88	0.00
Stream 5 (waste)	84.51	84.51	0.00
Stream 9 + resin (waste)	119.30	119.30	0.00
Stream 12	0.00	929.20	3100.00
Wash water UF	10.00	10.00	5.00
Membranes	0.07	0.07	0.25

Table S9. Equipment required for the whole process. Equipment types plus specifications used in the Aspen Plus® simulations and the resulting costs are listed

Process step	Equipment	Equipment type	Specifications	Cost [US$ piece^{-1}]	Source
Biocatalyst preparation	Mixing tank	Vertical process vessel	50 L, 0.35/0.52 (D/H)<br>SS316	4,800	Aspen
	Agitator	Portable direct drive agitator	0.5 kW<br>SS316	1,800	Aspen
Biosynthesis	Mixing tank	Vertical process vessel	50 L, 0.35/0.52 (D/H)<br>SS316	4,800	Aspen
	Agitator	Portable direct drive agitator	1 kW<br>SS316	2,500	Aspen
	Colum reactor	Jacketed vertical process vessel	30 L, 0.22/0.8 (D/H)<br>SS316<br>full, 5 barg	8,100	Aspen
Extraction/Stripping	Mixing tank extraction (6x)	Vertical process vessel	75 L, 0.415/0.555 (D/H)<br>SS316	5,400	Aspen
	Agitator extraction (6x)	Portable direct drive agitator	1 kW<br>SS316	2,500	Aspen
	Settle tank extraction (6x)	Vertical process vessel	75 L, 0.317/0.95 (D/H)<br>SS316	5,300	Aspen
	Mixing tank stripping (3x)	Vertical process vessel	45 L, 0.35/0.47 (D/H)<br>SS316	4,700	Aspen
	Agitator stripping (3x)	Portable direct drive agitator	1 kW<br>SS316	2,500	Aspen
	Settle tank stripping (3x)	Vertical process vessel	45 L, 0.267/0.8 (D/H)<br>SS316	4,900	Aspen
Borate removal	Column	Jacketed vertical process vessel	110 L, 0.3/1.56 (D/H)<br>SS316	9,800	Aspen
Ultrafiltration	Ultrafiltration		Used for the ultrafiltration of the aqueous phase and the concentrated retentate	4,000	
Nanofiltration	Mixing tank	Vertical process vessel	200 L, 0.48/1.1 (D/H)<br>SS316	7,100	Aspen
	Agitator	Portable direct drive agitator	2 kW,<br>SS316	3,000	Aspen
	Nanofiltration			187,000	
Concentration	Evaporation vessel 2-GG	Vertical process vessel	200 L, 0.48/1.1 (D/H)<br>SS316	7,100	Aspen
	Heating coil 2-GG	Electric immersion tank heater	20 kW<br>SS304	1,300	Aspen
	Evaporator Glycerol	Agitated falling film evaporator	2.5 m²<br>SS316	190,200	Aspen
Condensation	Coil cond 2-GG	Bare pipe immersion coil-heating/cooling	1 m²<br>SS316	1,100	Aspen

	Coil cond Glycerol	Bare pipe immersion coil-heating/cooling	10 m²<br>SS316	9,000	Aspen
	Storage vessel H_20	Cone bottom storage bin	3,500 L, 1.56/1.835 (D/H)<br>SS316	39,200	Aspen
Storage	Tank 2-GG	Bare pipe immersion coil-heating/cooling	35 L, 0.28/0.57 (D/H)<br>SS316	5,800	Aspen
	Coil cool 2-GG	Cone bottom storage bin	0.5 m²<br>SS316	610	Aspen
	Tank Glycerol	Bare pipe immersion coil-heating/cooling	250 L, 0.55/1.05 (D/H)<br>SS316	8,400	Aspen
	Coil cool Glycerol	Cone bottom storage bin	1 m²<br>SS316	1,100	Aspen

Table S10. Overview of the total capital investment

Position	Costs [€]
Equipment costs	502,012
Total plant direct costs	1,255,031
Total plant indirect costs	627,515
Other costs	301,207
Fixed capital investment	2,685,765
Working capital	134,288
Total capital investment	2,820,054

Table S11. Overview of the operation costs

Type	Description	Costs [€ a^{-1}]
Material costs	Biosynthesis	18,781
	Extraction/stripping	514,045
	Ultrafiltration	1,163
	Borate removal	17,910
	Diafiltration	14,928
Labor costs	Salaries	425,063
	Labor burden	340,050
Waste treatment	Water waste	250
	Aqueous waste	30,820
	Hazard waste	21,202
	Solid waste	15
Utilities	Electricity	70,940
	Cooling water	4,108
Other operation costs	Insurance	26,858
	Maintenance	52,711
Sum		1,538,842
Overhead costs		76,942
Total operation costs		1,615,784

Table S12. Overview of the discounted cash flow assessment

Year	Revenue product [€]	Revenue recovered material [€]	Total revenue [€]	Operation costs [€]	Depreciaction [€]	Remaining Process value [€]	Net revenue [€]	Losses [€]	Income [€]	Tax [€]	Net cashflow[€]	Discounted value [€]
1	1 915 283	9 008	1 924 291	1 615 784	537 153	2 148 612	-228 646		-228 646	0	308 507	280 460.9
2	1 953 588	9 189	1 962 777	1 648 100	537 153	1 611 459	-222 476	-228 646	-451 122	0	314 677	260 063.7
3	1 992 660	9 372	2 002 033	1 681 062	537 153	1 074 306	-216 182	-451 122	-667 304	0	320 971	241 150.0
4	2 032 513	9 560	2 042 073	1 714 683	537 153	537 153	-209 763	-667 304	-877 067	0	327 390	223 611.8
5	2 073 164	9 751	2 082 915	1 748 977	537 153	0	-203 215	-877 067	-1 080 283	0	333 938	207 349.1
6	2 114 627	9 946	2 124 573	1 783 956	0	0	340 617	-1 080 283	-739 666	0	340 617	192 269.2
7	2 156 919	10 145	2 167 064	1 819 635	0	0	347 429	-739 666	-392 237	0	347 429	178 286.0
8	2 200 058	10 348	2 210 406	1 856 028	0	0	354 378	-392 237	-37 860	0	354 378	165 319.7
9	2 244 059	10 555	2 254 614	1 893 149	0	0	361 465	-37 860	323 605	97 082	264 383	112 124.4
10	2 288 940	10 766	2 299 706	1 931 012	0	0	368 694	0	368 694	110 608	258 086	99 503.3
11	2 334 719	10 981	2 345 700	1 969 632	0	0	376 068	0	376 068	112 820	263 248	92 266.7
12	2 381 413	11 201	2 392 614	2 009 025	0	0	383 590	0	383 590	115 077	268 513	85 556.4
13	2 429 042	11 425	2 440 467	2 049 205	0	0	391 261	0	391 261	117 378	273 883	79 334.1
14	2 477 622	11 653	2 489 276	2 090 189	0	0	399 087	0	399 087	119 726	279 361	73 564.4
15	2 527 175	11 886	2 539 061	2 131 993	0	0	407 068	0	407 068	122 121	284 948	68 214.3
16	2 577 718	12 124	2 589 843	2 174 633	0	0	415 210	0	415 210	124 563	290 647	63 253.2
17	2 629 273	12 367	2 641 639	2 218 126	0	0	423 514	0	423 514	127 054	296 460	58 653.0
18	2 681 858	12 614	2 694 472	2 262 488	0	0	431 984	0	431 984	129 595	302 389	54 387.3
19	2 735 495	12 866	2 748 362	2 307 738	0	0	440 624	0	440 624	132 187	308 437	50 431.9
20	2 790 205	13 124	2 803 329	2 353 893	0	0	449 436	0	449 436	134 831	314 605	46 764.1
21	2 846 009	13 386	2 859 395	2 400 970	0	0	458 425	0	458 425	137 528	320 898	43 363.1
22	2 902 930	13 654	2 916 583	2 448 990	0	0	467 594	0	467 594	140 278	327 316	40 209.4
23	2 960 988	13 927	2 974 915	2 497 970	0	0	476 945	0	476 945	143 084	333 862	37 285.1
24	3 020 208	14 205	3 034 413	2 547 929	0	0	486 484	0	486 484	145 945	340 539	34 573.4
25	3 080 612	14 490	3 095 102	2 598 888	0	0	496 214	0	496 214	148 864	347 350	32 059.0
											Sum [€]	2 820 053,5

References

(1) Franceus, J.; Ubiparip, Z.; Beerens, K.; Desmet, T. Engineering of a Thermostable Biocatalyst for the Synthesis of 2-*O*-Glucosylglycerol. *ChemBioChem* **2021**, *22*, 2777–2782, DOI 10.1002/cbic.202100192.

(2) Goedl, C.; Sawangwan, T.; Mueller, M.; Schwarz, A.; Nidetzky, B. A High-Yielding Biocatalytic Process for the Production of 2-*O*-(α-D-Glucopyranosyl)-*sn*-Glycerol, a Natural Osmolyte and Useful Moisturizing Ingredient. *Angew. Chemie - Int. Ed.* **2008**, *47*, 10086–10089, DOI 10.1002/anie.200803562.

(3) Al Ghatta, A.; Wilton-Ely, J. D. E. T.; Hallett, J. P. From Sugars to FDCA: A Techno-Economic Assessment Using a Design Concept Based on Solvent Selection and Carbon Dioxide Emissions. *Green Chem.* **2021**, 1716–1733, DOI 10.1039/d0gc03991h.

(4) Karimi Alavijeh, M.; Meyer, A. S.; Gras, S. L.; Kentish, S. E. Simulation and Economic Assessment of Large-Scale Enzymatic N-Acetyllactosamine Manufacture. *Biochem. Eng. J.* **2020**, *154*, 107459, DOI 10.1016/j.bej.2019.107459.

(5) Swart, L. J.; Petersen, A. M.; Bedzo, O. K. K.; Görgens, J. F. Techno-Economic Analysis of the Valorization of Brewers Spent Grains: Production of Xylitol and Xylo-Oligosaccharides. *J. Chem. Technol. Biotechnol.* **2021**, *96*, 1632–1644, DOI 10.1002/jctb.6683.

(6) Zhang, Y.; Brown, T. R.; Hu, G.; Brown, R. C. Techno-Economic Analysis of Monosaccharide Production via Fast Pyrolysis of Lignocellulose. *Bioresour. Technol.* **2013**, *127*, 358–365, DOI 10.1016/j.biortech.2012.09.070.

(7) Dubbink, G. H. C.; Geverink, T. R. J.; Haar, B.; Koets, H. W.; Kumar, A.; van den Berg, H.; van der Ham, A. G. J.; Lange, J. P. Furfural to FDCA: Systematic Process Design and Techno-Economic Evaluation. *Biofuels, Bioprod. Biorefining* **2021**, 1–10, DOI

10.1002/bbb.2204.

(8) Turton, R.; Bailie, R. C.; Whiting, W. B.; Shaeiwitz, J. A.; Bhattacharyya, D. *Analysis, Synthesis, and Design of Chemical Processes*, 4th ed.; Pearson Education: Upper Saddler River, NJ, US, 2012.

(9) Bedzo, O. K. K.; Mandegari, M.; Görgens, J. F. Comparison of Immobilized and Free Enzyme Systems in Industrial Production of Short-Chain Fructooligosaccharides from Sucrose Using a Techno-Economic Approach. *Biofuels, Bioprod. Biorefining* **2019**, *13*, 1274–1288, DOI 10.1002/bbb.2025.

(10) Vaňková, K.; Onderková, Z.; Antošová, M.; Polakovič, M. Design and Economics of Industrial Production of Fructooligosaccharides. *Chem. Pap.* **2008**, *62*, 375–381, DOI 10.2478/s11696-008-0034-y.

(11) Karamerou, E.; Parsons, S.; McManus, M.; Chuck, C. Using Techno-Economic Modelling to Determine the Minimum Cost Possible for a Microbial Palm Oil Substitute. *Biotechnol. Biofuels* **2020**, 1–19, DOI 10.21203/rs.3.rs-43577/v1.

(12) Kazi, F. K.; Patel, A. D.; Serrano-Ruiz, J. C.; Dumesic, J. A.; Anex, R. P. Techno-Economic Analysis of Dimethylfuran (DMF) and Hydroxymethylfurfural (HMF) Production from Pure Fructose in Catalytic Processes. *Chem. Eng. J.* **2011**, *169*, 329–338, DOI 10.1016/j.cej.2011.03.018.

(13) Kruschitz, A.; Peinsipp, L.; Pfeiffer, M.; Nidetzky, B. Continuous Process Technology for Glucoside Production from Sucrose Using a Whole Cell-Derived Solid Catalyst of Sucrose Phosphorylase. *Appl. Microbiol. Biotechnol.* **2021**, *105*, 5383–5394, DOI 10.1007/s00253-021-11411-x.

(14) Kruschitz, A.; Nidetzky, B. Removal of Glycerol from Enzymatically Produced 2-α-D-

Glucosyl-Glycerol by Discontinuous Diafiltration. *Sep. Purif. Technol.* **2020**, *241*, 116749, DOI 10.1016/j.seppur.2020.116749.
